# Multiple aspects of the selfing syndrome of the morning glory *Ipomoea lacunosa* evolved in response to selection: A Qst‐Fst comparison

**DOI:** 10.1002/ece3.5329

**Published:** 2019-06-13

**Authors:** Joanna L. Rifkin, Irene T. Liao, Allan S. Castillo, Mark D. Rausher

**Affiliations:** ^1^ Department of Biology Duke University Durham North Carolina

**Keywords:** floral morphology, *Ipomoea*, mating system, natural selection, selfing syndrome, self‐pollination

## Abstract

The frequent transition from outcrossing to selfing in flowering plants is often accompanied by changes in multiple aspects of floral morphology, termed the “selfing syndrome.” While the repeated evolution of these changes suggests a role for natural selection, genetic drift may also be responsible. To determine whether selection or drift shaped different aspects of the pollination syndrome and mating system in the highly selfing morning glory *Ipomoea lacunosa*, we performed multivariate and univariate Qst‐Fst comparisons using a wide sample of populations of *I. lacunosa* and its mixed‐mating sister species *Ipomoea cordatotriloba*. The two species differ in early growth, floral display, inflorescence traits, corolla size, nectar, and pollen number. Our analyses support a role for natural selection driving trait divergence, specifically in corolla size and nectar traits, but not in early growth, display size, inflorescence length, or pollen traits. We also find evidence of selection for reduced herkogamy in *I. lacunosa*, consistent with selection driving both the transition in mating system and the correlated floral changes. Our research demonstrates that while some aspects of the selfing syndrome evolved in response to selection, others likely evolved due to drift or correlated selection, and the balance between these forces may vary across selfing species.

## 1 INTRODUCTION

The evolutionary transition from outcrossing to self‐pollination has occurred repeatedly in flowering plants (Barrett, 2002). This transition is typically accompanied by reductions in several traits distinct from the mechanism of mating system change: highly selfing species produce smaller flowers with less nectar, less scent, and lower pollen‐to‐ovule ratios than their outcrossing relatives (Goodwillie et al., 2010; Ornduff, 1969; Sicard & Lenhard, 2011). These features of highly selfing species are termed, by analogy with biotic pollination syndromes, the “selfing syndrome.” In addition to floral traits, selfing species often exhibit rapid growth and development compared to outcrossers and are more frequently annuals (Lloyd, 1992; Snell & Aarssen, 2005). Furthermore, although floral display size includes both the number of flowers and the size of those flowers (Goodwillie, Ritland, & Ritland, 2006), flower number, and inflorescence size in selfing species have been less studied than flower or petal size. Finally, the flowers of selfing species differ from those of outcrossing species not only in individual traits but also in the allometric relationships and correlations between traits (Fornoni, Ordano, Pérez‐Ishiwara, Boege, & Domínguez, 2016; Rosas‐Guerrero, Quesada, Armbruster, Pérez‐Barrales, & Smith, 2010; Vallejo‐Marín, Walker, Friston‐Reilly, Solis‐Montero, & Igic, 2014). Because selfing species are generally derived from outcrossing relatives (Igic & Busch, 2013; Sicard & Lenhard, 2011), it can be assumed that the outcrossing species more closely represents the ancestral state and that selfing‐syndrome changes are derived.

The reasons for these trait reductions remain unclear. While selection favoring the spread of selfing itself is theoretically well supported through the automatic advantage (Fisher, 1941) and reproductive assurance (Busch & Delph, 2012), there has been little effort to determine whether reductions in floral traits that do not directly affect selfing rate result from natural selection or from the accumulation of mutations through genetic drift. Both explanations are plausible. On the one hand, the repeated evolution of the selfing syndrome represents a convergent evolutionary response to a change in mating system, and convergent evolution can often indicate similar selective pressures (Losos, 2011; Stern, 2013). Several types of selection have been proposed to drive selfing‐syndrome evolution. For example, because costly floral displays are no longer needed to attract pollinators in highly selfing species, selection could favor reallocation of resources away from these displays to other fitness‐enhancing functions, such as higher fruit‐to‐flower and seed‐to‐ovule ratios (Goodwillie et al., 2010). Similarly, because less pollen is needed for self‐fertilization and as a reward for pollen‐feeding insects, selection could reallocate resources used to produce pollen to other functions (Goodwillie et al., 2010). Alternatively, reduced floral size and display size may be favored because they reduce attractiveness to herbivores (McCall & Irwin, 2006; Sicard & Lenhard, 2011). Finally, selection may often favor faster development in highly selfing species because they tend to grow in marginal habitats (Lloyd, 1992; Snell & Aarssen, 2005).

By contrast, genetic drift is also a plausible explanation for the evolution of selfing‐syndrome traits. Without the necessity of attracting insect pollinators, selfing plants no longer experience purifying selection to maintain display traits and may accumulate mutations that reduce floral size, pollen production, nectar production, and display size through genetic drift (Duncan & Rausher, 2013a). Moreover, self‐pollination increases homozygosity, which reduces effective population size and increases linkage disequilibrium, thereby increasing the potential effects of genetic drift (Charlesworth & Wright, 2001; Pollak & Sabran, 1992). Random mutations in coding sequence generally have an adverse effect on protein function (Eyre‐Walker & Keightley, 2007), and, in genes controlling floral size, mutations that alter protein function often cause floral size reduction (Krizek & Fletcher, 2005). Relatedly, inbreeding depression can manifest as generally reduced size as a result of the accumulation of mildly deleterious variants (Charlesworth & Charlesworth, 1987). For these reasons, genetic drift causing the accumulation of mutations that reduce floral traits should not be ignored as a possible cause of the general pattern of reductions in the selfing syndrome in any individual species. Yet to our knowledge, there have been few investigations into the role of selection in shaping selfing‐syndrome traits that do not directly affect selfing rate. Two recent studies—on corolla size in the morning glories *Ipomoea lacunosa* and *Ipomoea cordatotriloba* (Duncan & Rausher, 2013a) and flower size in *Collinsia heterophylla* (Strandh, Jönsson, Madjidian, Hansson, & Lankinen, 2017)—indicated a role for natural selection in the divergence of both floral and reproductive traits. Neither, however, included elements of the selfing syndrome outside of floral dimensions, such as nectar, pollen, or growth traits.

A related but distinct question is whether selection favors the initial spread of self‐pollination. Typically, the evolution of increased selfing, whether for reproductive assurance or because of an automatic transmission advantage (Barrett, 2002), is thought to be driven by selection, which is required to overcome the inbreeding depression that is common in outcrossing species (Busch & Delph, 2012). Studies of selection on self‐pollination have estimated current levels of inbreeding depression, pollen discounting, and seed discounting (Busch & Delph, 2012; Husband & Schemske, 1996; Layman, Fernando, Herlihy, & Busch, 2017; Rausher, Augustine, & Vanderkooi, 1993), and whether selfing is favored in conditions of pollen or pollinator limitation (Briscoe Runquist, Geber, Pickett‐Leonard, & Moeller, 2017; Fishman & Willis, 2008; Gervasi & Schiestl, 2017; Moeller & Geber, 2005; Roels & Kelly, 2011; Strandh et al., 2017; Toräng et al., 2017). However, we know of fewer direct demonstrations of overall selection favoring selfing, although a recent study in *Leavenworthia* found molecular signatures consistent with positive selection on the S‐locus (Herman & Schoen, 2016). Thus, while the forces favoring the spread of self‐pollination are well characterized theoretically and empirically, it is important to confirm rather than assume that selection has in fact favored the adoption of selfing in individual cases.

Qst‐Fst comparisons between quantitative traits and neutral markers offer a method for differentiating between selection and drift as possible causes for population or species divergence (Leinonen, McCairns, O'Hara, & Merilä, 2013). When the genes responsible for individual traits are not known, either because the traits are polygenic, because the system is a nonmodel species, or both, molecular signatures of selection (Nielsen, 2005) cannot be used to detect selection on divergent traits. In these situations, a Qst‐Fst approach, which uses phenotypic measurements to estimate the differentiation in loci underlying quantitative traits of interest and compares that differentiation to neutral markers, can discriminate between selection and drift as explanations for phenotypic divergence.

In this investigation, we apply Qst‐Fst approaches to determine the extent to which natural selection contributed to the evolution of selfing‐syndrome traits and of self‐pollination in *I. lacunosa*. This study greatly expands upon the initial investigation by Duncan and Rausher (Duncan & Rausher, 2013a) in several ways. First, it includes selfing‐syndrome traits besides floral size, such as nectar production, pollen production, and display size (flower number) and thus constitutes the first attempt to determine whether reductions in these traits in highly selfing species are also caused by selection. Second, we estimate genetic variance more precisely with measurements taken from individuals of known parentage in the greenhouse rather than wild individuals. Third, this study examines traits that are not traditionally considered part of the selfing syndrome, but are thought to correlate with high selfing rates (inflorescence size and growth rate). We determine whether these traits differ between the species and whether they differ in the directions predicted by the expectations of the selfing syndrome—that is, that floral traits should be generally reduced in the selfing species and growth rate possibly increased. Finally, this study includes a broader array of populations and a much larger number of neutral loci and dramatically changes our previous estimates of Fst.

In addition to exploring how selection has shaped floral changes that follow the mating system transition, we use the Qst‐Fst approach to detect selection for increased selfing itself. In self‐compatible morning glories, selfing rate is controlled by herkogamy (anther‐stigma separation), a quantitative trait known to be evolutionarily labile in many species (Chang & Rausher, 1998; Duncan & Rausher, 2013a; Opedal, Bolstad, Hansen, Armbruster, & Pélabon, 2017). We therefore use the Qst‐Fst framework to determine whether selection favored reduced herkogamy in *I. lacunosa*.

## 2 MATERIALS AND METHODS

### 2.1 Study system

*Ipomoea lacunosa* and *I. cordatotriloba* are weeds in the series *Batatas* of the genus *Ipomoea* (Convolvulaceae; USDA & NRCS, 2017). A recent comprehensive phylogenetic analysis of this series indicates that they are sister species (Muñoz‐Rodríguez et al., 2018). The two species have overlapping distributions in North America: *I. lacunosa* is found in the Eastern United States from Florida to Canada and west to Texas, and *I. cordatotriloba* occurs from Mexico to North Carolina (for map, see Rifkin, Castillo, Liao, & Rausher, 2019). We have observed both species growing intertwined in the same habitat.

**Figure 1:**
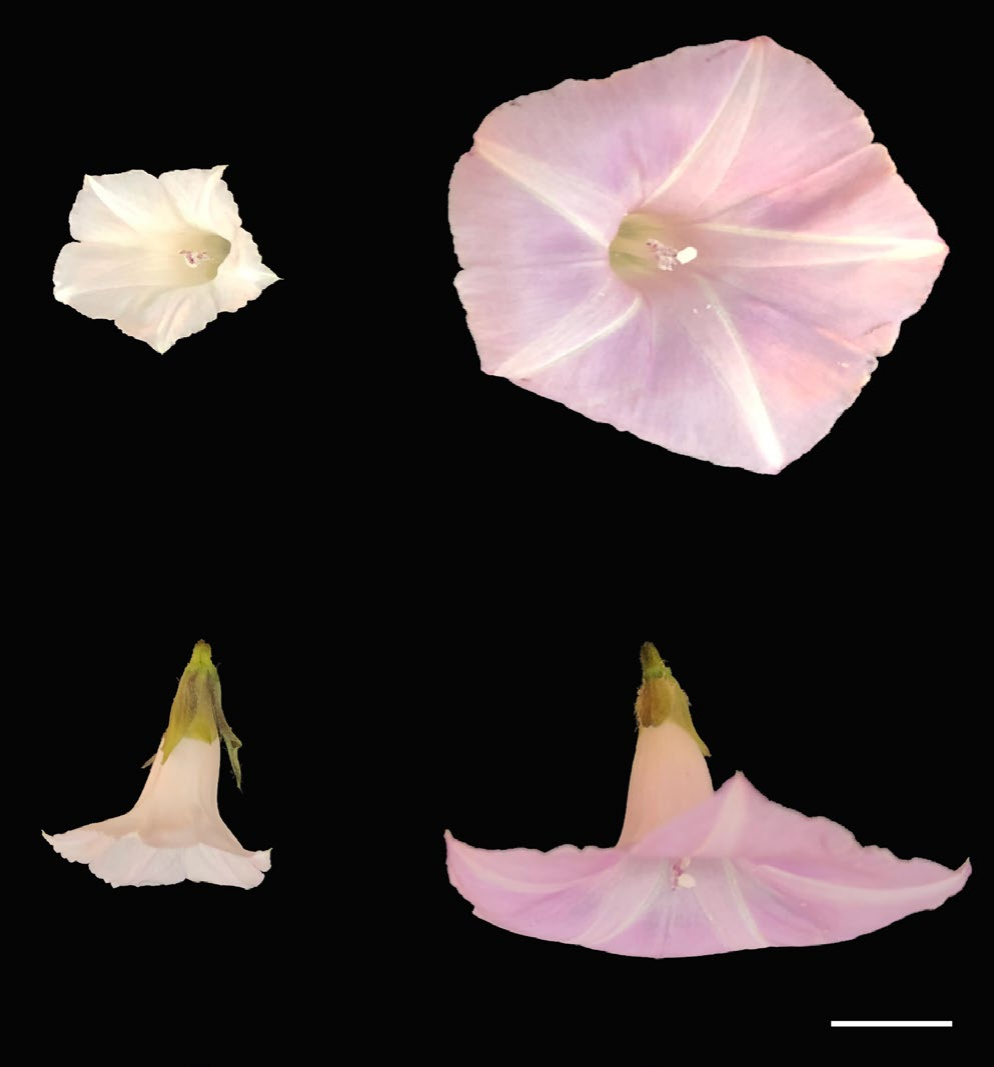
Floral morphological differences between *Ipomoea lacunosa* (left) and *Ipomoea cordatotriloba* (right). *Ipomoea cordatotriloba* flowers are larger, more open, and generally exhibit greater herkogamy. Scale bar = 1 cm

Both species are self‐compatible. However, *I. lacunosa* is highly selfing (selfing rate > 0.95), while *I. cordatotriloba*'s selfing rate varies widely among populations and averages around 0.5 (Duncan & Rausher, 2013a). The highly selfing *I. lacunosa* produces small, white flowers with less pollen and less nectar than *I. cordatotriloba*, which produces larger purple flowers (Figure 1; McDonald, Hansen, McDill, & Simpson, 2011; Rifkin, 2017; Rifkin et al., 2019). Incomplete crossing barriers separate the two species, but artificial hybrids can be produced, and natural hybrids have been reported (Abel & Austin, 1981; Diaz, Schmiediche, & Austin, 1996; Duncan & Rausher, 2013b; Rifkin, 2017).

### 2.2 Samples and plant culture

For this study, we used plants covering a wide geographic range obtained from the Rausher Lab's field collections, an accession from the Baskin Lab, and accessions from the USDA's GRIN seed bank (Rifkin et al., 2019). Our samples included 33 *I. cordatotriloba* accessions from 13 sites and 31 *I. lacunosa* accessions from 12 sites. From each site, we used no more than three accessions (a complete list of the accessions used can be found on the Dryad Digital Repository). One selfed offspring from all but three of these accessions was genotyped for the Fst analysis, which is thus based on 61 individuals. For Qst estimation, we grew two selfed offspring from each of these accessions and three additional accessions that were not genotyped but were from the same region (Austin, TX) as a genotyped accession that never produced flowers. A total of 114 individuals were thus phenotyped. The limited per‐population sample numbers may affect our estimates of within‐species population variation, but as our focus is between‐species differentiation and given the magnitude of between‐species differentiation (see below), this should not affect our conclusions. In addition, with a large number of genetic loci, accurate estimates of Fst can be obtained from even a small number of individuals (Willing, Dreyer, & van Oosterhout, 2012).

To grow plants for genotyping and phenotyping, seeds were scarified and planted in four‐inch pots in Fafard 4P soil and maintained in a growth room under 16‐hr days at 25.6°C (78°F). After 4 weeks, conditions were changed to 12‐hr days at 18.3°C (65°F) to trigger flowering. When flower buds appeared, plants were moved to the Duke Greenhouse and grown under the following conditions: 12‐hr days at 23–26°C (74–80°F), 12‐hr nights at 16–19°C (61–67°F), 65% relative humidity, and 700µmol s s^−1^/cm^2^. The plants were allowed to acclimate for 2 weeks before any measurements were taken.

### 2.3 Phenotypic measurements

We included measurements of nine characters in our analysis: the traditional selfing‐syndrome characters corolla length, corolla width, nectar volume, nectar sugar concentration, and pollen number, as well as characters associated with early growth (length of first three internodes at day 21) and display size (total flowers per day, length of inflorescence from stem to flower base, number of flowers on inflorescence). To determine if selection favored increased selfing in *I. lacunosa*, we measured herkogamy (the degree to which anthers are positioned below the stigma), a major determinant of selfing rate in these species (Duncan & Rausher, 2013a). Herkogamy was measured as the number of anthers that were below the stigma: in highly outcrossing populations, the style extends above the anthers, while in selfing populations, anthers touch the stigma (Duncan & Rausher, 2013a). For comparison, we also measured three vegetative traits that we have no reason to believe are associated with selfing: sepal length, leaf length/width ratio, and leaf dissection.

Early growth measurements of the first three internodes (cotyledons to first true leaf, first true leaf to second true leaf, and second true leaf to third true leaf) were taken with a rule on day 21 after planting. After plants were moved to the greenhouse, floral measurements were performed between 8:00 a.m. and 12:00 p.m. Because flowers remain open for less than a day, it is not necessary to standardize flower age. For most individuals, three flowers were measured on different days. Corolla length and width, inflorescence length, and style length were measured using a digital caliper (Mitutoyo Digimatic CD6″ CS).

Nectar volume and sugar concentration were quantified from flowers that had been covered overnight to prevent evaporation. The day before a flower opened, the bud was capped with a plastic straw covered with parafilm. The following morning, all nectar was extracted from the base of the flower with 2 µl microcapillary tubes (Drummond Scientific). The height of the nectar in the tube was measured with the digital caliper. Because each tube is 32 mm long and holds 2 µl in total, this measurement was converted to volume with the formula *V* = (2 µl * height of nectar in tube)/32 mm. Nectar sugar concentration was quantified by expelling all the nectar from the microcapillary tube onto a Master‐53M ATAGO refractometer. The refractometer was standardized with water at the start of each day's measurements. Because refractometer readings are often imprecise with low volumes, two sugar concentration measurements were taken: undiluted nectar and nectar diluted with 2.5µl water. The relationship between sugar concentration measured as weight/weight (w/w) and as measured by mol/L or mg/ml is nonlinear (Bolten, Feinsinger, Baker, & Baker, 1979). Therefore, we used data on the relationship between w/w and mol/L for sucrose solutions from the CRC Handbook of Chemistry and Physics to convert mol/L into mg/ml values (Rumble, 2018, p. 5–132). Specifically, we converted the mol/L values in the table to mg/ml values by multiplying the mol/L by the molecular weight of sucrose (342.2964 g/mol). We then calculated a regression equation to convert w/w into mg/ml: mg/ml = 0.0524(w/w)^2^ + 9.6554(w/w) + 1.3904 (*R* ^2^ = 0.9999). For nectar diluted with 2.5µl water, the diluted sugar concentration was first converted into mg/ml, then multiplied by the ratio: (actual nectar amount + 2.5µl)/actual nectar amount. The diluted and undiluted nectar sugar concentration values in mg/ml were averaged to produce the nectar sugar concentration used in our analyses.

Pollen production, measured as pollen per ovule, was quantified by removing anthers the day before anthesis, allowing them to dry overnight in an open microcentrifuge tube, and resuspending them in 500 µl 70% ethanol. Pollen was quantified by manually counting all pollen grains in a 100 µl aliquot from each sample under a dissecting microscope, multiplying by five (500 µl/100 µl), and dividing by 4 (the fixed number of ovules in both species; McDonald et al., 2011).

Leaf length and width and degree of dissection were scored for three mature leaves on each individual. Dissection was scored visually on a five‐point scale as follows: 0, all leaves entire with no visible lobing; 0.25, some leaves slightly lobed with three points visible but no indentation; 0.5, some leaves entire and some leaves lobed; 0.75, most leaves lobed; 1, all leaves lobed and indented (figure available at Dryad Digital Repository). This reflected both the degree to which individual leaves were dissected and the extent to which all of a plant's leaves were dissected or not. Sepal length was measured with a digital caliper when nectar measurements were taken. For each individual, mean floral measurements were calculated in R. All subsequent analyses were performed on the averaged measurements for each individual. For multivariate analyses, we used the sum of the internodes rather than each individual internode as our measure of early growth.

### 2.4 Character divergence

To determine which traits differed between the two species, we performed a nested ANOVA using the “Fit Model” platform in JMP Pro 13 (SAS Institute, 2017). For each trait, we analyzed a model in which species was a fixed effect, while site nested within species and female parent (accession) nested within site were random effects, using restricted estimation maximum likelihood fits. Six individuals that never produced flowers were excluded from analysis. Pollen per ovule was log‐transformed for analysis; all other traits were approximately normally distributed and were therefore not transformed. Measurements on two selfed offspring per female parent (accession) provided the error effect. To correct for multiple comparisons, we applied a Benjamini and Hochberg false discovery rate correction (Benjamini & Hochberg, 1995). As a descriptive measure, we also calculated the relative divergence between species means as the absolute value of the difference between the two species means divided by whichever species mean was larger for the trait in question.

### 2.5 SNP calling

To estimate Fst, we identified SNPs from leaf transcriptomes. Total RNA was extracted from a single young leaf (0.5–2.0 cm in length) using a modified TRI Reagent (Sigma‐Aldrich) protocol that included an additional TRI Reagent:chloroform cleanup step, addition of glycogen, and three ethanol washes. RNA was resuspended in 30µl RNAse‐free water. We assessed RNA quality using a 2,200 TapeStation system (Agilent Technologies), and all samples displayed an RNA integrity score of at least 7. RNA sequencing libraries were generated using a KAPA Stranded mRNA‐Seq kit (KAPA Biosystems). For sequencing, libraries were multiplexed with NEBNext Multiple Oligos for Illumina (New England BioLabs) and quality‐checked with a Bioanalyzer Agilent High Sensitivity DNA kit (Agilent Technologies) and a Qubit Fluorometer (Thermo‐Fisher Scientific). Samples were pooled and sequenced using three lanes of the Illumina HiSeq 4000 v4 platform with 150 bp paired‐end reads at the Duke Sequencing and Genomic Technologies Shared Resource. The extraction protocol is available on GitHub (https://github.com/joannarifkin/IpomoeaQstFst).

SNPs were called and filtered using a modified version of the GATK best practices for RNASeq (Van der Auwera et al., 2013). We aligned reads to the *I. lacunosa* draft genome assembly with STAR 2‐pass (Dobin & Gingeras, 2015). Alignment files were cleaned, marked for duplicate reads, assigned read groups, and sorted using PicardTools (http://broadinstitute.github.io/picard). Reads extending into introns were truncated using Split “n” Trim. We used the GATK Joint Genotyper in ‐erc GVCF mode to call SNPs and hard‐filtered based on Fisher Strand Bias < 30, quality‐by‐depth < 2, minimum depth 10 reads, SNP clustering, and at least 60 individuals (out of 61 total) called. The resulting 66,729 SNPs were then coded as either synonymous, nonsynonymous, or noncoding using an APL (Iverson, 1962) script written by one of the authors (MDR) and an annotated draft *I. lacunosa* genome generated by our laboratory. A total of 27,079 synonymous SNPs were identified and used for estimating Fst. All scripts are available on GitHub (https://github.com/joannarifkin/IpomoeaQstFst).

Fst can be affected by the manner in which SNPs are ascertained (Bhatia, Patterson, Sankararaman, & Price, 2013). Ideally, only SNPs that were polymorphic in the ancestral population before speciation should be included, but this is often not possible to determine. We therefore examined the effects of two different ascertainment protocols. In the first protocol, we used all synonymous SNPs that were polymorphic in at least one of the two species (*N* = 27,079). With this protocol, Fst may be biased upward because the set of SNPs may include mutations that have arisen since divergence of the two species as well as ancestral variants that have been lost in one lineage but not the other (which is likely in a highly selfing species). In the second protocol, we used only SNPs that were polymorphic in both species. This approach may yield an estimate of Fst that is biased downward because it excludes ancestral SNPs that have become fixed in one species. To control for these effects, we calculated Fst from two datasets: all synonymous SNPs (“All SNPs,” *N* = 27,079) and synonymous SNPs that were polymorphic in both species (“Shared SNPs,” *N* = 2,352). We also repeated the “All SNPs” estimate with LD‐pruned SNPs separated by either 20kb or 40kb (consistent with the distance of LD decay in *I. lacunosa*, (Rifkin et al., 2019)), but it did not alter our estimates and we therefore report the estimate based on the full sample of SNPs.

### 2.6 Qst‐Fst comparison

The Qst‐Fst test asks whether Qst is significantly greater than Fst. If divergence in a quantitative trait is due to genetic drift, then it is expected that Qst = Fst. By contrast, if divergence was caused by selection, then it is expected that Qst > Fst (Leinonen et al., 2013). For each of the traits that exhibit significant divergence between species, we asked whether Qst is significantly greater than Fst.

### 2.7 Multivariate Qst‐Fst comparison

We performed a multivariate Qst‐Fst comparison on selfing‐syndrome traits that were significantly diverged between species (early growth, flowers per day, inflorescence length, corolla length and width, herkogamy, nectar volume and concentration, and pollen number) according to the methods of Martin et al. (Chapuis, Martin, & Goudet, 2008; Martin, Chapuis, & Goudet, 2008). We adapted R code made available by the authors (http://www.isem.univ-montp2.fr/fr/personnel/equipes/metapopulations/martin-guillaume.index/) by adding steps to round matrices to 12 decimal places to avoid loss of symmetry via loss of significance and to transform to positive definite by changing null eigenvalues to very small eigenvalues. This test relies on the property that if covariance matrices are evolving neutrally, then while they may vary considerably, they should on average be proportional to each other with the expected coefficient of proportionality *ρ*_Exp_ = 2Fst * (1 − Fst) in outcrossing species, or *ρ*_Exp_ = Fst * (1 − Fst) in a highly selfing species (Martin et al., 2008; Phillips, Whitlock, & Fowler, 2001; Rogers & Harpending, 1983). If the observed coefficient of proportionality between the G‐matrices significantly differs from this expectation, then that suggests the action of nonneutral processes. We chose to use the proportionality constant for a highly selfing species, as one of our species is highly selfing and the other moderately selfing; however, using the constant for an outcrossing species does not change our results.

To estimate the covariance matrices, we performed a MANOVA using accession means of traits transformed to Gaussian distributions and normalized to the means with “Species” as a factor. We estimated the coefficient of proportionality *ρ*_Obs_ between the G‐matrices G_A_ and G_S_, where G_A_ is the accession‐level G‐matrix and is equal to MS_A_ (mean squares at the accession level, based on the residual sum of squares divided by degrees of freedom) and G_S_ is between‐species level G‐matrix, estimated from MS_B_ (mean squares at the species level) and MS_A_. A detailed description of these calculations can be found in the supplementary methods and scripts are available at https://github.com/joannarifkin/IpomoeaQstFst.

To estimate the neutral coefficient of proportionality Fst * (1 − Fst), we computed Weir‐Cockerham estimates of Fst and its 95% confidence intervals using the **hierfstat** package in R at the species level across all individuals (Goudet, 2005; de Meeûs & Goudet, 2007; Weir & Cockerham, 1984). A confidence interval could not be generated for our coefficient of proportionality estimate because there is only one degree of freedom in the two‐species comparison. Therefore, we instead estimated a distribution of values for the observed coefficient of proportionality (*ρ*_Obs_) by resampling accessions within each species with replacement over 1,000 bootstrap replicates. We then compared the 95% confidence interval of this distribution of the observed coefficient of proportionality (*ρ*_Obs_) to the 95% confidence interval of the expected coefficient of proportionality (*ρ*_Exp_).

### 2.8 Univariate Qst‐Fst comparisons

We also performed Qst‐Fst comparisons on the traits individually to gain more insight into which traits might be driving the multivariate changes most strongly. Fst was calculated using Weir and Cockerham estimates in **hierfstat** as described above (Goudet, 2005; de Meeûs & Goudet, 2007; Weir & Cockerham, 1984). We also applied Hudson's estimator (Hudson, Slatkin, & Maddison, 1992; Keinan, Mullikin, Patterson, & Reich, 2007); because we found similar values of Fst with both methods, we report only the Weir‐Cockerham results.

Because our analysis of characters revealed little evidence of population structure within each species, we ignored populations when calculating Qst. Qst is generally defined by the formula

(1) $$\text{Qst}=\sigma_{\text{BetwSp}}^{2}/\left(\sigma_{\text{BetwSp}}^{2}+2V_{A}\right)$$

where *σ* ^2^_BetwSp_ is the between‐species component of variance and *V_A_* is the additive genetic variance for the trait (Whitlock, 2008). For each character, we estimated the Between‐Species variance component, *σ* ^2^_BetwSp_, and the Between‐Accession variance component, *σ* ^2^_BetwAcc_ from an ANOVA in which species was the top‐level effect, accession was nested within species, and there were two replicated individuals per accession. We then calculated Qst as

(2) $$\text{Qst}*=\sigma_{\text{BetwSp}}^{2}/\left(\sigma_{\text{BetwSp}}^{2}+2\sigma_{\text{BetwSp}}^{2}\right)$$

Because *I. lacunosa* is highly selfing, and *I. cordatotriloba* has a mixed mating system with a selfing rate of approximately 0.5, the between‐accessions component of variance will estimate the total genetic variance, *V_G_*, in *I. lacunosa*. In *I. cordatotriloba*, it will estimate a quantity between V_A _and V_G_. In either case, *σ* ^2^_BetwAcc_ > *V_A_*, making Qst* an underestimate of the true value of Qst, and thus making the Qst‐Fst test conservative because the difference between Qst* and Fst is an underestimate of the true difference.

We employed two methods to compare our estimates of Qst and Fst. First, we applied a standard nonparametric bootstrapping approach, which we refer to as “standard bootstrap” analysis. Using an APL program written by MDR, 1,000 bootstrap values of Qst and Fst were generated by randomly choosing accessions (Female Parent) with replacement. For each sample, we calculated Fst and Qst as described above, then calculated the difference D = Qst − Fst. From these values, we calculated the proportion of D values that were ≤0. We performed this analysis only on characters that showed a significant difference between species in the ANOVA described above.

Second, we used Whitlock and Gilbert's method for parametric bootstrapping of Qst in unbalanced half‐sib designs (Gilbert & Whitlock, 2014, 2015; Whitlock & Gilbert, 2012; Whitlock & Guillaume, 2009). Because the distribution of Qst for neutral traits varies depending on demographic traits, this approach uses the estimate of Fst to simulate a distribution based on the expectation that under neutrality

(3) $$\sigma_{\text{BetwSp}}^{2}\approx 2\;\bar{F}\;\text{st}\left(V_{A}\right)/\left(1-\bar{F}\;\text{st}\right)$$

We used the tool QstFstComp in the R package **QstFstComp** (Gilbert & Whitlock, 2014) in “half.sib.dam” mode (despite the name, it can be set to model any level of relatedness between siblings) with two populations, 1,000 simulations, and “dam.offspring.relatedness” set to 1 to reflect selfed offspring.

## 3 RESULTS

### 3.1 Differences between species

Using a nested ANOVA, we found little evidence for population differentiation within species (a table can be found on the Dryad Digital Repository): after a false discovery rate correction, the population effect was not significant for any of the traits. This does not necessarily mean there is no real population differentiation, only that we were not able to detect it, probably because we scored a maximum of three accessions per population. Variation among accessions within populations was significant for several traits (the early growth traits internode 1 and internode 3 elongation, the floral morphology traits of corolla length and width, and the vegetative trait of leaf shape), indicating the presence of genetic variation in these traits. Although other traits did not show a significant accession effect, we again do not claim there is no genetic variation for these traits, only that we did not have the power to detect it. In the Qst calculations, we assume that such variation is reflected in the *σ* ^2^_BetwAcc_ component of variance.

Generally, traits typically included in the selfing syndrome (corolla length and width, nectar volume, nectar sugar concentration, and pollen number) were significantly reduced in *I. lacunosa*, even after correction for multiple comparisons (Table 1). Herkogamy also differed significantly, as expected, with more anthers tending to be below the stigma in *I. cordatotriloba*. In contrast, the early growth trait of internode length on day 21 after germination was significantly increased in *I. lacunosa*, indicating faster early growth. In addition, number of flowers produced per day was significantly greater and inflorescence length significantly shorter in *I. lacunosa*. All of these differences, with the possible exception of the increase in number of flowers per day, are in the expected direction for a highly selfing species. The increase in number of flowers per day could reflect either an increase in display size (counter to expectation) or an investment in rapid reproduction in a marginal environment or relaxation of selection against geitonogamy (both consistent with expectation). Two of the three vegetative traits we examined (leaf length/width ratio and sepal length) did not differ significantly between the species, although *I. cordatotriloba* did produce more dissected leaves.

**Table 1:** Trait means, standard deviations and nested ANOVA results

Trait (measurement)	*Ipomoea lacunosa* Mean (*SD*) *N*	*Ipomoea cordatotriloba* Mean (*SD*) *N*	Nested ANOVA, species effect (F ratio, *p* value)	Relative divergence	Qst
Internode 1 (mm)*	13.37 (3.71) 60	5.67 (2.30) 49	93.4718, *p* < 0.0001	0.58	0.738
Internode 2 (mm)*	13.35 (6.78) 60	6.22 (2.95) 49	26.6351, *p* < 0.0001	0.53	0.537
Internode 3 (mm)*	52.20 (20.50) 60	20.80 (14.80) 49	49.5274, *p* < 0.0001	0.60	0.559
Flowers per day*	4.09 (1.79) 60	1.87 (1.21) 52	27.4305, *p* < 0.0001	0.54	0.671
Flowers on inflorescence	2.07 (0.80) 60	2.29 (1.16) 52	0.9087, *p* = 0.3502	0.10	NA
Inflorescence length (mm)*	9.59 (3.58) 60	19.85 (11.49) 52	15.1556, *p* = 0.0008	0.52	0.355
Corolla length (mm)*	20.00 (1.40) 60	32.09 (4.14) 52	105.0461, *p* < 0.0001	0.38	0.837
Corolla width*	15.09 (1.35) 60	34.44 (5.63) 52	157.7737, *p* < 0.0001	0.56	0.892
Nectar volume (µl)*	0.77 (0.36) 60	3.33 (1.19) 49	63.6928, *p* < 0.0001	0.77	0.753
Nectar sugar concentration (mg/ml)*	228.06 (42.38) 60	361.40 (46.63) 49	101.6822, *p* < 0.0001	0.35	0.824
Pollen grains per ovule*	157.79 (49.38) 60	213.00 (74.43) 52	8.1749, *p* = 0.0103	0.29	0.400
Leaf length/width	1.10 (0.09) 60	1.11 (0.11) 52	0.1167, *p* = 0.7361	<0.01	NA
Leaf dissection*	0.11 (0.21) 60	0.29 (0.28) 52	6.5780, *p* = 0.0183	0.60	NA
Sepal length (mm)	11.16 (0.72) 60	11.17 (1.20) 52	0.0087, *p* = 0.9266	<0.01	NA
Herkogamy*	0.28 (0.71) 60	3.35 (1.47) 51	74.3468, *p* < 0.0001	0.92	0.766

Note

Relative divergence was calculated as the absolute value of the difference between the two species means divided by whichever species mean was larger for the trait in question. NA indicates Qst was not calculated because divergence was not significant. All trait values have been rounded to two significant figures, but calculations were performed with unrounded values.

* Traits where species difference was significant at the 0.05 level after a false discovery rate correction.

### 3.2 Qst‐Fst analysis

The estimate of Fst from all synonymous SNPs (Fst = 0.442, 95% CI 0.437, 0.447) was higher than Fst estimated using only shared polymorphic SNPs (Fst = 0.390, 95% CI 0.375, 0.405). A higher Fst estimate from “all” than from “shared” SNPs is consistent with the expected biases introduced by the two ascertainment protocols.

### 3.3 Multivariate analysis

If divergence of a set of traits is neutral in a highly selfing species, the between‐species and within‐species G‐matrices will be proportional, with the coefficient of proportionality *ρ*_Exp_ = Fst * (1 − Fst). This corresponds to *ρ*_Exp_ = 0.2466 and *ρ*_Exp_ = 0.2379 for all synonymous SNPs and shared SNPs, respectively. The corresponding covariance matrices can be found on the Dryad Digital Repository.

The maximum likelihood estimate of the observed coefficient of proportionality *ρ*_Obs_ was 2.093. One thousand bootstrap replicates produced a 95% confidence interval of (0.6836, 7.0654) around our maximum likelihood estimate. This does not overlap the 95% confidence interval for the neutral expectation of Fst(1 − Fst) derived from either all SNPs (0.2460, 0.2472) or shared SNPs (0.2344, 0.2409). The difference between our observed *ρ* and the neutral expectation of *ρ* is consistent with nonneutral trait divergence between these two species.

### 3.4 Univariate analyses

To identify which individual traits were subject to selection, we performed a univariate Qst‐Fst analysis on each selfing‐syndrome trait that differed between the two species. For herkogamy, the estimated Qst was 0.766, which is substantially higher than either estimate of Fst. This difference was significant in the standard bootstrap analysis for both all SNPs and shared SNPs (*p* < 0.0001 for both). It was also significant in the parametric bootstrap for the analysis using Fst estimated from shared SNPs (*p* = 0.03) and of borderline significance (*p* = 0.053) in the parametric bootstrap analysis using Fst estimated from all SNPs. We interpret these results to be consistent with selection having caused the evolution of reduced herkogamy in *I. lacunosa*.

**Figure 2:**
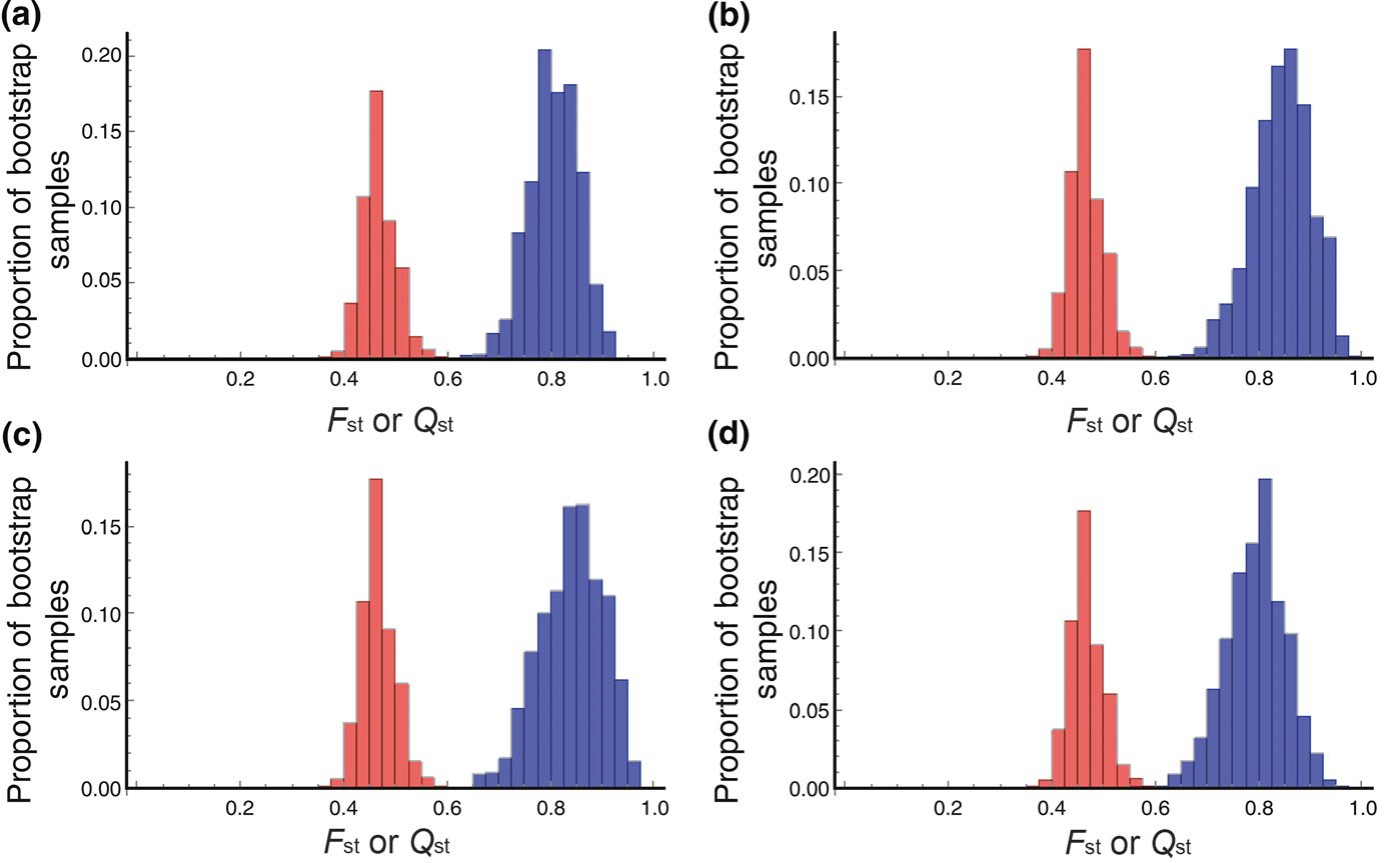
Bootstrap histograms for Fst (Red) and Qst (Blue) for the four characters showing significant Qst‐Fst differences in the standard bootstrap analyses using all SNPs. (a) Corolla length. (b) Corolla width. (c) Nectar volume. (d) Sugar concentration

**Figure 3:**
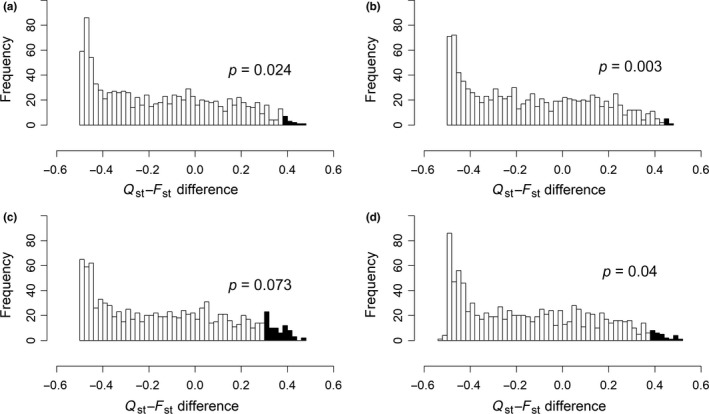
Distribution of differences between resampled neutral Qst and resampled neutral Fst values in the parametric bootstrap analysis using all SNPs. Shaded portion indicates resampled differences above observed Qst‐Fst difference. (a) Corolla length. (b) Corolla width. (c) Nectar volume. (d) Sugar concentration

In the standard bootstrap analysis, four selfing‐syndrome traits exhibited significant Qst – Fst differences for both Fst estimates (Table 2, Figure 2). These traits include both floral dimensions (corolla length and width) and both nectar traits (volume and sugar concentration). In the parametric bootstrap analysis, three and four of these same traits exhibited significance at *p* < 0.05 when using the Fst values corresponding to all and shared SNPs, respectively (Table 2, Figure 3). The fourth trait, nectar volume, was borderline significant (*p* = 0.073) with all SNPs. Although one trait lost significance in the parametric bootstrap with one set of SNPs, this test is known to have low power if there are only two populations (Gilbert & Whitlock, 2015). Overall, the analyses suggest that the divergence between the two species in floral morphology was due to selection, and that that selection acted chiefly on floral dimensions, nectar volume, and nectar sugar concentration. In contrast, although early growth, pollen and display traits are also components of the selfing syndrome, we did not find evidence of direct selection on these traits.

**Table 2:** Qst‐Fst differences in simple and parametric bootstrap

Trait (measurement)	*p* value (simple bootstrap, all SNPs)	*p* value (simple bootstrap, shared SNPs)	*p* value (parametric bootstrap, all SNPs)	*p* value (parametric bootstrap, shared SNPs)
Internode 1 (mm)	0.092	0.094	0.081	0.054
Internode 2 (mm)	0.276	0.299	0.292	0.218
Internode 3 (mm)	0.156	0.162	0.227	0.199
Flowers per day	0.108	0.112	0.187	0.151
Inflorescence length (mm)	0.837	0.836	0.444	0.355
Corolla length (mm)	0.001*	0.001*	0.024*	0.007*
Corolla width (mm)	0.001*	0.001*	0.003*	0.004*
Nectar volume (µl)	0.001*	0.001*	0.073	0.041*
Nectar sugar concentration (mg/ml)	0.001*	0.001*	0.04*	0.01*
Pollen grains per ovule	0.789	0.796	0.396	0.344

Abbreviations: All SNPs, all polymorphic SNPs; Shared SNPs, only SNPs polymorphic in both species.

* Tests that are significant at the *p* < 0.05 level.

## 4 DISCUSSION

### 4.1 Evolution of increased selfing in *I. lacunosa*

Mating system transitions, particularly transitions from outcrossing to selfing, are among the most common evolutionary changes in plants (Barrett, 2002). Increases in selfing rate can influence processes that can affect fitness, such as the magnitude of inbreeding depression experienced, the reduction in pollen transmission to other plants because of pollen discounting, increased reproductive assurance, and loss of heterozygosity (Jarne & Charlesworth, 1993). Given these effects on fitness, it seems likely that in most cases, changes in selfing rate are driven by natural selection. Our results are consistent with this expectation in indicating that selection was responsible for the evolution of reduced herkogamy, and thus higher selfing rates, in *I. lacunosa*. Since reproductive assurance and the automatic advantage are well‐supported theoretical bases for the evolution of selfing, and since as a weedy annual *I. lacunosa* may have particularly benefited from reproductive assurance, it is possible that they played a role in this transition. At this point, however, we do not know with any certainty the factors responsible for selection favoring selfing in this species.

### 4.2 The role of selection on selfing‐syndrome traits

The floral and life‐history changes that constitute the selfing syndrome are potentially distinct from morphological and physiological changes that directly affect selfing, such as reduced herkogamy and loss of self‐incompatibility. Although these associated floral and life‐history changes have been observed in a wide range of taxa, and various selective explanations have been proposed for them, there have been few attempts to determine whether selection is indeed responsible for these changes (Duncan & Rausher, 2013a; Strandh et al., 2017). Genetic drift is also a plausible explanation for these differences. Selfing plants experience relaxed selection to attract pollinators, and their reduced effective population size makes them more susceptible to the effects of drift (Charlesworth & Wright, 2001; Pollak & Sabran, 1992). Random mutations and inbreeding depression are likely to cause floral size reduction (Charlesworth & Charlesworth, 1987; Eyre‐Walker & Keightley, 2007). For these reasons, determining whether selection is responsible for these changes is valuable for understanding how and why the selfing syndrome evolved.

We have demonstrated that *I. lacunosa* has evolved the classic selfing‐syndrome traits of reduced flower size, reduced nectar production, and reduced pollen production, as well as inflorescence structure and early growth differences that may be associated with weediness and annuality. Moreover, we have shown that divergence between species in this suite of traits was likely driven by natural selection, based on multivariate Qst‐Fst analysis. From our univariate analyses, we find support for selection having played a role in reducing corolla size and nectar volume and sugar concentration.

By contrast, other selfing‐syndrome traits, including several that are strongly diverged between the two species (e.g., inflorescence length, flowers produced each day, early growth, and pollen production), showed no evidence that divergence was caused by selection. There are three explanations for this result: (a) our Qst‐Fst test may not have been powerful enough to detect selection on these traits; (b) these traits may truly not have experienced divergent selection, but diverged due to genetic drift; and (c) divergence in these traits may have been caused by correlated selection on other traits.

Studies in other species suggest that indirect selection on correlated traits may account for divergence in traits that did not display evidence of selection. In general, floral traits have been found to be moderately to strongly correlated, both phenotypically and genetically (Bernacchi & Tanksley, 1997; Fishman, Beardsley, Stathos, Williams, & Hill, 2014; Fishman, Kelly, & Willis, 2002; Georgiady, Whitkus, & Lord, 2002; Goodwillie et al., 2006; Lin & Ritland, 1997; Slotte, Hazzouri, & Stern, 2012). For example, previous studies have found evidence for a tradeoff between flower size and flower number (Ashman & Majetic, 2006; Sargent, Goodwillie, Kalisz, & Ree, 2007). If such a tradeoff exists in *Ipomoea*, the detected selection favoring decreased flower size could have resulted in the observed increase in flower number in *I. lacunosa*. Similarly, if pollen production is correlated with flower size, as is the case in *Capsella* and *Mimulus* (Fishman et al., 2002; Slotte et al., 2012), the observed selection for reduced flower size may have led to less pollen in *I. lacunosa* as a correlated response. This is particularly likely in a selfing species. Selfing species tend to show increased integration among traits (Vallejo‐Marín et al., 2014), which may reflect stronger correlations due to increased linkage disequilibrium (Fornoni et al., 2016). Therefore, correlated selection remains a plausible explanation for divergence in traits which did not show evidence of selection.

While we find that divergence in four selfing‐syndrome characters was likely caused by selection, and can thus rule out genetic drift in these traits, we are not able to determine from our data whether direct or indirect selection acted on each of these characters. Although to our knowledge this issue has not been examined, it is possible that Qst‐Fst differences could reflect correlated rather than direct responses to selection. It is thus possible that, say, direct selection to reduce corolla length and width could also reduce nectar production, if reduction in floral dimensions caused a correlated reduction in nectary size. An artificial selection experiment in *Eichhornia paniculata* found evidence consistent with this when selection for increased flower size also led to increased nectar volume, suggesting underlying genetic correlations (Worley & Barrett, 2000). However, we suspect that a correlated response of this type detectable from a Qst‐Fst analysis would require a strong genetic correlation between floral dimensions and nectary size. Identifying the genetic bases of these traits and relationships among them may allow us to distinguish between direct selection, correlated selection, and drift.

As is traditional, we have assumed that selfing‐syndrome characters evolved after the evolution of increased selfing and did not themselves contribute directly to an increase in selfing rate. However, to the extent that the reductions in floral characters in *I. lacunosa* render flowers less attractive to pollinators, one consequence of selection on these characters may be reduced outcrossing and thus increased selfing. It is thus possible that these selfing‐syndrome characters are not so much a consequence of increased selfing but a contributor to it. Further experiments will be required to determine the extent to which these characters may have evolved because they increased selfing rates.

In addition to traditional selfing‐syndrome characters, we also examined early growth. Researchers have long observed an association between selfing, weediness, and an annual life history, and selfing plants develop faster at several life stages (Fishman et al., 2014; Snell & Aarssen, 2005). We found that stem elongation during the first 3 weeks after germination was significantly faster in *I. lacunosa*, consistent with the evolution of more rapid growth. The early growth differences might lead to the expectation of more general vegetative differences in response to a more marginal habitat, and the other vegetative trait that had diverged, leaf dissection, is associated with patterns of local water availability (Nicotra et al., 2011). However, our Qst‐Fst analysis does not suggest that early growth divergence was caused by natural selection. Measurements of physiological traits such as specific leaf area might reveal further differences in vegetative characteristics, especially since *I. lacunosa*'s range extends further north than *I. cordatotriloba*'s (USDA & NRCS, 2017; Wilson, Thompson, & Hodgson, 1999), but these vegetative divergences can probably be regarded as distinct from the selfing syndrome: the geographic ranges of the two species overlap such that both likely experience similar pressures on vegetative growth in much of their range, and the one study that we are aware of testing the relationship between physiology and selfing rate found no relationship (Ivey, Dudley, Hove, Emms, & Mazer, 2016). In addition, the magnitude of morphological divergence is far greater for floral than for vegetative traits. In most species, floral and vegetative traits are not tightly correlated (Ashman & Majetic, 2006), and indeed, we found no evidence of divergence in a flower‐adjacent vegetative trait (sepal length, known to be variable in this group of species: Austin, 1978).

### 4.3 Power and limitations of Qst‐Fst analysis

In our analyses, the standard bootstrap approach detected higher levels of significance than the parametric bootstrap approach. This raises the question of which approach provides a better picture of selection on these traits. Unlike parametric bootstrap, the standard bootstrap approach does not account for the possibility that populations may by chance drift in a similar direction (Whitlock, 2008; Whitlock & Guillaume, 2009). In our analysis, however, we compare only two “populations,” that is, the two species, rendering this problem moot. Another issue, raised by O'Hara and Merilä (2005), is that standard bootstrap methods tend to underestimate Qst, especially for Qst > 0.7, as is true for the four significant traits. That renders our approach conservative, suggesting that tests based on standard bootstrapping may be valid in our case. While the parametric bootstrap approach tends to give more reliable estimates of Qst (O'Hara & Merilä, 2005), it also has low power when the number of “populations” is small (Gilbert & Whitlock, 2015), as in our study. Low power may thus explain why *p*‐values for the four significant traits in the standard bootstrap analysis are lower than in the parametric analysis.

A more important caveat is that all current methods for Qst‐Fst comparisons rely on assumptions that natural populations may not satisfy (Gilbert & Whitlock, 2015; Leinonen et al., 2013; Whitlock, 2008). Aside from more general issues, such as the assumption of exclusively additive gene action (Cubry, Scotti, Oddou‐Muratorio, & Lefèvre, 2017; Santure & Wang, 2008), three aspects of *I. lacunosa* and *I. cordatotriloba* specifically violate these assumptions: the degree of divergence between the species, the between‐species comparison, and the nature of *I. lacunosa* as a highly selfing species.

Simulation studies have found that Qst‐Fst studies are generally more reliable when Fst is below 0.2 (Whitlock & Guillaume, 2009). While the original microsatellite estimate of Fst (0.04; Duncan & Rausher, 2013b) was within this range, a larger sample of neutral SNPs yielded an estimate of over 0.4, which is considerably higher. High Fst is difficult to differentiate from even very high Qst values (Whitlock, 2008). The Lewontin–Krakauer distribution in parametric Qst‐Fst comparisons also provides inaccurate estimates of neutral Qst at high values of Fst (Whitlock & Guillaume, 2009), and when very few populations are used (e.g., when two species or subspecies are compared), the *χ* ^2^ distribution used for resampling neutral Qst is skewed such that it is difficult to differentiate the resampled neutral estimate of Qst from the actual Qst value (Figure 2; Gilbert & Whitlock, 2015). While these issues primarily reduce the power to detect Qst‐Fst differences, we nevertheless managed to detect significant differences despite this power reduction. Finally, differences between subspecies or species are likely to contain more nonadditive genetic differences (Whitlock & Gilbert, 2012). Dominance increases Qst and the variance of Qst relative to purely additive inheritance when selection is acting, but does not increase the likelihood of false positives from neutrally diverging traits (Cubry et al., 2017; Santure & Wang, 2008). Thus, the increased nonadditive differences in highly diverged populations may reduce power but should not increase the likelihood of false positives in estimates of Qst‐Fst difference.

Selfing affects our Qst‐Fst comparisons in two ways: it shapes the estimates generated of Qst and it may alter the relationship between Qst and Fst. The selfing breeding design to generate our families may provide more accurate Qst estimates for selfing species (Goudet & Büchi, 2006), so it is probably not a cause for concern. The history of inbreeding, on the other hand, may have complex effects on the estimation of Qst. Inbreeding can inflate the variance of Qst (Cubry et al., 2017), although its effects are generally less dramatic than those of dominance (Cubry et al., 2017; Santure & Wang, 2008). Finally, the effects of purging of deleterious alleles and of inbreeding depression are not accounted for in a Qst‐Fst framework (Gilbert & Whitlock, 2015).

Overall, these sources of bias are more likely to reduce than to inflate the magnitude of Qst‐Fst differences. Interspecific differentiation and a history of inbreeding both reduce the likelihood of false positives by raising Fst. Dominance does not generally increase the likelihood of false positives. Both parametric and standard bootstrapping are conservative in differentiating Qst and Fst (O'Hara & Merilä, 2005). Since multiple aspects of our methods (estimate of Qst, bootstrapping, parametric bootstrapping) are conservative or expected to lack power in diverged, selfing species, we argue that our analysis is conservative overall and the evidence we identify for selection is compelling. Inbreeding and self‐pollination are common in plants and may be associated with different selective regimes (Allendorf, Hohenlohe, & Luikart, 2010; Barrett, 2002). To better understand phenotypic evolution in inbreeding populations, quantitative genetic theory should develop methods for Qst‐Fst comparisons that can more precisely account for this variation in demographic history or interface with existing demographic inference models to differentiate neutral divergence from drift.

## 5 CONCLUSIONS

Our findings partially support the general expectation that natural selection drives the evolution of selfing‐syndrome traits. In particular, we found that this was true for reductions in floral size and nectar traits. Surprisingly, however, we did not find that selection caused reduced pollen production, a key feature of the selfing syndrome in *I. lacunosa* and other highly selfing species. This complicates and expands upon the conclusions of Duncan and Rausher (2013a), in which both selfing‐syndrome traits examined diverged in response to selection. In addition, we found no evidence that selection modified other characters typically associated with highly selfing plants, such as display size and growth rates. In the absence of selection, changes in these traits may have been caused largely by genetic drift or by correlated selection that was too weak to detect. Distinguishing between these two possibilities will require additional experiments examining the genetic correlation structure of selfing‐syndrome traits.

## CONFLICT OF INTEREST

None declared.

## AUTHOR CONTRIBUTIONS

JLR, ITL, and MDR designed the study, analyzed the data, and wrote the manuscript. ASC, JLR, ITL collected the specimens. JLR and ITL performed phenotypic measurements (JLR: all traits except sepal length and nectar traits; ITL: sepal length and nectar traits). ASC and ITL prepared RNA libraries. JLR and ASC performed the SNP calling.

## Data Availability

Sequence read data: Dryad Digital Repository (https://doi.org/10.5061/dryad.f6qb7c5). Phenotypic data, a figure demonstrating leaf shapes, the site‐ and accession‐level effects for the ANOVA of phenotypic data, covariance matrices, and a full list of the accessions used with latitude and longitude have been uploaded to the Dryad Digital Repository (https://doi.org/10.5061/dryad.7vs53cr). Nucleic acid extraction protocol, phenotypic data, SNP‐calling scripts, and Qst‐Fst scripts are available at https://github.com/joannarifkin/IpomoeaQstFst.

## References

[ece35329-bib-0001] Abel, W. E. , & Austin, D. F. (1981). Introgressive hybridization between *Ipomoea trichocarpa* and *Ipomoea lacunosa* (Convolvulaceae). Bulletin of the Torrey Botanical Club, 108(2), 231–239. 10.2307/2484902

[ece35329-bib-0002] Allendorf, F. W. , Hohenlohe, P. A. , & Luikart, G. (2010). Genomics and the future of conservation genetics. Nature Reviews Genetics, 11(10), 697–709. 10.1038/nrg2844 20847747

[ece35329-bib-0003] Ashman, T.‐L. , & Majetic, C. J. (2006). Genetic constraints on floral evolution: A review and evaluation of patterns. Heredity, 96(5), 343–352. 10.1038/sj.hdy.6800815 16598191

[ece35329-bib-0004] Austin, D. F. (1978). The *Ipomoea batatas* complex ‐ I. Taxonomy. Bulletin of the Torrey Botanical Club, 105(2), 114–129. 10.2307/2484429

[ece35329-bib-0005] Barrett, S. C. (2002). The evolution of plant sexual diversity. Nature Reviews Genetics, 3(4), 274–284. 10.1038/nrg776 11967552

[ece35329-bib-0006] Benjamini, Y. , & Hochberg, Y. (1995). Controlling the false discovery rate: A practical and powerful approach to multiple testing. Journal of the Royal Statistical Society: Series B (Methodological), 57(1), 289–300. 10.1111/j.2517-6161.1995.tb02031.x

[ece35329-bib-0007] Bernacchi, D. , & Tanksley, S. (1997). An interspecific backcross of *Lycopersicon esculentum* × *L. hirsutum*: Linkage analysis and a QTL study of sexual compatibility factors and floral traits. Genetics, 14850, 861–877. 10.1093/genetics/147.2.861 PMC1208205 9335620

[ece35329-bib-0008] Bhatia, G. , Patterson, N. , Sankararaman, S. , & Price, A. L. (2013). Estimating and interpreting FST: The impact of rare variants. Genome Research, 23(9), 1514–1521. 10.1101/gr.154831.113 23861382 PMC3759727

[ece35329-bib-0009] Bolten, A. B. , Feinsinger, P. , Baker, H. G. , & Baker, I. (1979). On the calculation of sugar concentration in flower nectar. Oecologia, 18, 301–304. 10.1007/BF00377434 28309767

[ece35329-bib-0010] Briscoe Runquist, R. D. , Geber, M. A. , Pickett‐Leonard, M. , & Moeller, D. A. (2017). Mating system evolution under strong pollen limitation: Evidence of disruptive selection through male and female fitness in *Clarkia xantiana* . The American Naturalist, 189(5), 549–563. 10.1086/691192 28410019

[ece35329-bib-0011] Busch, J. W. , & Delph, L. F. (2012). The relative importance of reproductive assurance and automatic selection as hypotheses for the evolution of self‐fertilization. Annals of Botany, 109(3), 553–562. 10.1093/aob/mcr219 21937484 PMC3278291

[ece35329-bib-0012] Chang, S. , & Rausher, M. D. (1998). Frequency‐dependent pollen discounting contributes to maintenance of a mixed mating system in the common morning glory *Ipomoea purpurea* . The American Naturalist, 152(5), 671–683. 10.2307/2463845 18811342

[ece35329-bib-0013] Chapuis, E. , Martin, G. , & Goudet, J. (2008). Effects of selection and drift on G matrix evolution in a heterogeneous environment: A multivariate Qst‐Fst test with the freshwater snail *Galba truncatula* . Genetics, 180(4), 2151–2161. 18854589 10.1534/genetics.108.092452 PMC2600948

[ece35329-bib-0014] Charlesworth, D. , & Charlesworth, B. (1987). Inbreeding depression and its evolutionary consequences. Annual Review of Ecology and Systematics, 18(1987), 237–268. 10.1146/annurev.es.18.110187.001321

[ece35329-bib-0015] Charlesworth, D. , & Wright, S. I. (2001). Breeding systems and genome evolution. Current Opinion in Genetics & Development, 11(6), 685–690. 10.1016/S0959-437X(00)00254-9 11682314

[ece35329-bib-0016] Cubry, P. , Scotti, I. , Oddou‐Muratorio, S. , & Lefèvre, F. (2017). Generalization of the Q ST framework in hierarchically structured populations: Impacts of inbreeding and dominance. Molecular Ecology Resources, 17(6), e76–e83. 28681534 10.1111/1755-0998.12693

[ece35329-bib-0017] de Meeûs, T. , & Goudet, J. (2007). A step‐by‐step tutorial to use HierFstat to analyse populations hierarchically structured at multiple levels. Infection, Genetics and Evolution, 7(6), 731–735. 10.1016/j.meegid.2007.07.005 17765664

[ece35329-bib-0018] Diaz, J. , Schmiediche, P. , & Austin, D. F. (1996). Polygon of crossability between eleven species of *Ipomoea*: Section Batatas (Convolvulaceae). Euphytica, 88(1979), 189–200. 10.1007/BF00023890

[ece35329-bib-0019] Dobin, A. , & Gingeras, T. R. (2015). Mapping RNA‐seq Reads with STAR. Current Protocols in Bioinformatics, 51, 11.14.1–11.14.9. 26334920 10.1002/0471250953.bi1114s51 PMC4631051

[ece35329-bib-0020] Duncan, T. M. , & Rausher, M. D. (2013a). Evolution of the selfing syndrome in *Ipomoea* . Frontiers in Plant Science, 4, 1–8. 10.3389/fpls.2013.00301 23950758 PMC3738867

[ece35329-bib-0021] Duncan, T. M. , & Rausher, M. D. (2013b). Morphological and genetic differentiation and reproductive isolation among closely related taxa in the *Ipomoea* series *Batatas* . American Journal of Botany, 100(11), 2183–2193. 24169430 10.3732/ajb.1200467

[ece35329-bib-0022] Eyre‐Walker, A. , & Keightley, P. D. (2007). The distribution of fitness effects of new mutations. Nature Reviews Genetics, 8, 610–618. 10.1038/nrg2146 17637733

[ece35329-bib-0023] Fisher, R. (1941). Average excess and average effect of a gene substitution. Annals of Human Genetics, 11(1), 53–63.

[ece35329-bib-0024] Fishman, L. , Beardsley, P. M. , Stathos, A. , Williams, C. F. , & Hill, J. P. (2014). The genetic architecture of traits associated with the evolution of self‐pollination in *Mimulus* . The New Phytologist, 205, 907–917. 25306861 10.1111/nph.13091

[ece35329-bib-0025] Fishman, L. , Kelly, A. J. , & Willis, J. H. (2002). Minor quantitative trait loci underlie floral traits associated with mating system divergence in *Mimulus* . Evolution, 56(11), 2138–2155. 10.1111/j.0014-3820.2002.tb00139.x 12487345

[ece35329-bib-0026] Fishman, L. , & Willis, J. H. (2008). Pollen limitation and natural selection on floral characters in the yellow monkeyflower, *Mimulus guttatus* . New Phytologist, 177(3), 802–810. 18005321 10.1111/j.1469-8137.2007.02265.x

[ece35329-bib-0027] Fornoni, J. , Ordano, M. , Pérez‐Ishiwara, R. , Boege, K. , & Domínguez, C. A. (2016). A comparison of floral integration between selfing and outcrossing species: A meta‐analysis. Annals of Botany, 117(2), 299–306. 26578721 10.1093/aob/mcv166 PMC4724042

[ece35329-bib-0028] Georgiady, M. , Whitkus, R. , & Lord, E. (2002). Genetic analysis of traits distinguishing outcrossing and self‐pollinating forms of currant tomato, *Lycopersicon pimpinellifolium* (Jusl.) Mill. Genetics, 344, 333–344. 10.1093/genetics/161.1.333 PMC1462093 12019247

[ece35329-bib-0029] Gervasi, D. D. L. , & Schiestl, F. P. (2017). Real‐time divergent evolution in plants driven by pollinators. Nature Communications, 8, 1–8. 10.1038/ncomms14691 PMC5424062 28291771

[ece35329-bib-0030] Gilbert, K. J. , & Whitlock, M. C. (2014). QstFstComp: Qst Fst comparisons with unbalanced half‐sib designs. R package version 0.2. 10.1111/1755-0998.12303 25042150

[ece35329-bib-0031] Gilbert, K. J. , & Whitlock, M. C. (2015). QST‐FST comparisons with unbalanced half‐sib designs. Molecular Ecology Resources, 15(2), 262–267. 25042150 10.1111/1755-0998.12303

[ece35329-bib-0032] Goodwillie, C. , Ritland, C. , & Ritland, K. (2006). The genetic basis of floral traits associated with mating system evolution in *Leptosiphon* (Polemoniaceae): An analysis of quantitative trait loci. Evolution, 60(3), 491–504. 10.1111/j.0014-3820.2006.tb01131.x 16637495

[ece35329-bib-0033] Goodwillie, C. , Sargent, R. D. , Eckert, C. G. , Elle, E. , Geber, M. A. , Johnston, M. O. , … Winn, A. A. (2010). Correlated evolution of mating system and floral display traits in flowering plants and its implications for the distribution of mating system variation. The New Phytologist, 185(1), 311–321. 10.1111/j.1469-8137.2009.03043.x 19807872

[ece35329-bib-0034] Goudet, J. (2005). HIERFSTAT, a package for R to compute and test hierarchical F‐statistics. Molecular Ecology Notes, 5, 184–186. 10.1111/j.1471-8286.2004.00828.x

[ece35329-bib-0035] Goudet, J. , & Büchi, L. (2006). The effects of dominance, regular inbreeding and sampling design on QST, an estimator of population differentiation for quantitative traits. Genetics, 172(2), 1337–1347. 16322514 10.1534/genetics.105.050583 PMC1456230

[ece35329-bib-0036] Herman, A. C. , & Schoen, D. J. (2016). Recent selection for self‐compatibility in a population of *Leavenworthia alabamica* . Evolution, 70(6), 1212–1224. 27139712 10.1111/evo.12937

[ece35329-bib-0037] Hudson, R. R. , Slatkin, M. , & Maddison, W. P. (1992). Estimation of levels of gene flow from DNA sequence data. Genetics, 132(2), 583–589. 1427045 10.1093/genetics/132.2.583 PMC1205159

[ece35329-bib-0038] Husband, B. C. , & Schemske, D. W. (1996). Evolution of the magnitude and timing of inbreeding depression in plants. Evolution, 50(1), 54–70. 10.1111/j.1558-5646.1996.tb04472.x 28568860

[ece35329-bib-0039] Igic, B. , & Busch, J. W. (2013). Is self‐fertilization an evolutionary dead end? The New Phytologist, 198(2), 386–397. 10.1111/nph.12182 23421594

[ece35329-bib-0040] Iverson, K. E. (1962). A programming language. Proceedings of the May 1–3, 1962, Spring Joint Computer Conference. ACM.

[ece35329-bib-0041] Ivey, C. T. , Dudley, L. S. , Hove, A. A. , Emms, S. K. , & Mazer, S. J. (2016). Outcrossing and photosynthetic rates vary independently within two *Clarkia* species: Implications for the joint evolution of drought escape physiology and mating system. Annals of Botany, 118(5), 897–905. 10.1093/aob/mcw134 PMC5055815 27443300

[ece35329-bib-0042] Jarne, P. , & Charlesworth, D. (1993). The evolution of the selfing rate in functionally hermaphrodite plants and animals. Annual Review of Ecology and Systematics, 24(1993), 441–466. 10.1146/annurev.es.24.110193.002301

[ece35329-bib-0043] Keinan, A. , Mullikin, J. C. , Patterson, N. , & Reich, D. (2007). Measurement of the human allele frequency spectrum demonstrates greater genetic drift in East Asians than in Europeans. Nature Genetics, 39(10), 1251–1255. 10.1038/ng2116 17828266 PMC3586588

[ece35329-bib-0044] Krizek, B. A. , & Fletcher, J. C. (2005). Molecular mechanisms of flower development: An armchair guide. Nature Reviews Genetics, 6(9), 688–698. 10.1038/nrg1675 16151374

[ece35329-bib-0045] Layman, N. C. , Fernando, M. T. R. , Herlihy, C. R. , & Busch, J. W. (2017). Costs of selfing prevent the spread of a self‐compatibility mutation that causes reproductive assurance. Evolution, 71(4), 884–897. 10.1111/evo.13167 28075480

[ece35329-bib-0046] Leinonen, T. , McCairns, R. J. S. , O'Hara, R. B. , & Merilä, J. (2013). Q(ST)‐F(ST) comparisons: Evolutionary and ecological insights from genomic heterogeneity. Nature Reviews Genetics, 14, 179–190. 10.1038/nrg3395 23381120

[ece35329-bib-0047] Lin, J. , & Ritland, K. (1997). Quantitative trait loci differentiating the outbreeding *Mimulus guttatus* from the inbreeding *M. platycalyx* . Genetics, 146, 1115–1121. 9215912 10.1093/genetics/146.3.1115 PMC1208039

[ece35329-bib-0048] Lloyd, D. G. (1992). Self‐ and cross‐fertilization in plants. II. The selection of self‐fertilization. International Journal of Plant Sciences, 153(3), 370–380. 10.1086/297041

[ece35329-bib-0049] Losos, J. B. (2011). Convergence, adaptation, and constraint. Evolution, 65(7), 1827–1840. 10.1111/j.1558-5646.2011.01289.x 21729041

[ece35329-bib-0050] Martin, G. , Chapuis, E. , & Goudet, J. (2008). Multivariate Qst‐Fst comparisons: A neutrality test for the evolution of the G matrix in structured populations. Genetics, 180(4), 2135–2149. 18245845 10.1534/genetics.107.080820 PMC2600947

[ece35329-bib-0051] McCall, A. C. , & Irwin, R. E. (2006). Florivory: The intersection of pollination and herbivory. Ecology Letters, 9(12), 1351–1365. 10.1111/j.1461-0248.2006.00975.x 17118009

[ece35329-bib-0052] McDonald, J. A. , Hansen, D. R. , McDill, J. R. , & Simpson, B. B. (2011). A phylogenetic assessment of breeding systems and floral morphology of North American *Ipomoea* (Convolvulaceae). Journal of the Botanical Research Institute of Texas, 5(1), 159–177.

[ece35329-bib-0053] Moeller, D. A. , & Geber, M. A. (2005). Ecological context of the evolution of self‐pollination in *Clarkia xantiana*: Population size, plant communities, and reproductive assurance. Evolution, 59(4), 786–799. 10.1554/04-656 15926689

[ece35329-bib-0054] Muñoz‐Rodríguez, P. , Carruthers, T. , Wood, J. R. I. , Williams, B. R. M. , Weitemier, K. , Kronmiller, B. , … Scotland, R. W. (2018). Reconciling conflicting phylogenies in the origin of sweet potato and dispersal to Polynesia. Current Biology, 28, 1–11. 10.1016/j.cub.2018.03.020 29657119

[ece35329-bib-0055] Nicotra, A. B. , Leigh, A. , Boyce, C. K. , Jones, C. S. , Niklas, K. J. , Royer, D. L. , & Tsukaya, H. (2011). The evolution and functional significance of leaf shape in the angiosperms. Functional Plant Biology, 38(7), 535–552. 10.1071/FP11057 32480907

[ece35329-bib-0056] Nielsen, R. (2005). Molecular signatures of natural selection. Annual Review of Genetics, 39, 197–218. 10.1146/annurev.genet.39.073003.112420 16285858

[ece35329-bib-0057] O'Hara, R. B. , & Merilä, J. (2005). Bias and precision in QST estimates: Problems and some solutions. Genetics, 171(3), 1331–1339. 16085700 10.1534/genetics.105.044545 PMC1456852

[ece35329-bib-0058] Opedal, Ø. H. , Bolstad, G. H. , Hansen, T. F. , Armbruster, W. S. , & Pélabon, C. (2017). The evolvability of herkogamy: Quantifying the evolutionary potential of a composite trait. Evolution, 71(6), 1–31. 10.1111/evo.13258 28440562

[ece35329-bib-0059] Ornduff, R. (1969). Reproductive biology in relation to systematics. Taxon, 18(2), 121–133. 10.2307/1218671

[ece35329-bib-0060] Phillips, P. C. , Whitlock, M. C. , & Fowler, K. (2001). Inbreeding changes the shape of the genetic covariance matrix in *Drosophila melanogaster* . Genetics, 158, 1137–1145. 11454762 10.1093/genetics/158.3.1137 PMC1461705

[ece35329-bib-0061] Pollak, E. , & Sabran, M. (1992). On the theory of partially inbreeding finite populations. III. Fixation probabilities under partial selfing when heterozygotes are intermediate in viability. Genetics, 131(4), 979–985. 1516824 10.1093/genetics/131.4.979 PMC1205107

[ece35329-bib-0062] Rausher, M. D. , Augustine, D. , & Vanderkooi, A. (1993). Absence of pollen discounting in a genotype of *Ipomoea purpurea* exhibiting increased selfing. Evolution, 47(6), 1688–1695. 28567997 10.1111/j.1558-5646.1993.tb01261.x

[ece35329-bib-0063] Rifkin, J. L. (2017). Population genetics, natural selection and genetic architecture of the selfing syndrome in the morning glory *Ipomoea lacunosa*. Durham, NC: Duke University.

[ece35329-bib-0064] Rifkin, J. L. , Castillo, A. S. , Liao, I. T. , & Rausher, M. D. (2019). Gene flow, divergent selection and resistance to introgression in two species of morning glories (Ipomoea). Molecular Ecology, 28, 1–21. 30451335 10.1111/mec.14945

[ece35329-bib-0065] Roels, S. A. B. , & Kelly, J. K. (2011). Rapid evolution caused by pollinator loss in *Mimulus guttatus* . Evolution, 65(9), 2541–2552. 10.1111/j.1558-5646.2011.01326.x 21884055 PMC5958604

[ece35329-bib-0066] Rogers, A. T. , & Harpending, H. C. (1983). Population structure and quantitative characters. Genetics, 203–227. 10.1093/genetics/105.4.985 PMC1202238 17246186

[ece35329-bib-0067] Rosas‐Guerrero, V. , Quesada, M. , Armbruster, W. S. , Pérez‐Barrales, R. , & Smith, S. D. W. (2010). Influence of pollination specialization and breeding system on floral integration and phenotypic variation in *Ipomoea* . Evolution, 65(2), 350–364. 10.1111/j.1558-5646.2010.01140.x 20874738

[ece35329-bib-0068] Rumble, J. (2018). CRC handbook of chemistry and physics (99th ed.). Boca Raton, FL: Taylor & Francis.

[ece35329-bib-0069] Santure, A. W. , & Wang, J. (2008). The joint effects of selection and dominance on the QST ‐ FST contrast. Genetics, 181(1), 259–276. 18984567 10.1534/genetics.108.097998 PMC2621174

[ece35329-bib-0070] Sargent, R. D. , Goodwillie, C. , Kalisz, S. , & Ree, R. H. (2007). Phylogenetic evidence for a flower size and number trade‐off. American Journal of Botany, 94(12), 2059–2062. 10.3732/ajb.94.12.2059 21636399

[ece35329-bib-0071] SAS Institute (2017). JMP Pro 13. Cary, NC: SAS Institute.

[ece35329-bib-0072] Sicard, A. , & Lenhard, M. (2011). The selfing syndrome: A model for studying the genetic and evolutionary basis of morphological adaptation in plants. Annals of Botany, 107(9), 1433–1443. 10.1093/aob/mcr023 21303786 PMC3108801

[ece35329-bib-0073] Slotte, T. , Hazzouri, K. , & Stern, D. (2012). Genetic architecture and adaptive significance of the selfing syndrome in *Capsella* . Evolution, 66(5), 1360–1374. 22519777 10.1111/j.1558-5646.2011.01540.x PMC5063048

[ece35329-bib-0074] Snell, R. , & Aarssen, L. W. (2005). Life history traits in selfing versus outcrossing annuals: Exploring the “time‐limitation” hypothesis for the fitness benefit of self‐pollination. BMC Ecology, 5, 2. 15707481 10.1186/1472-6785-5-2 PMC553978

[ece35329-bib-0075] Stern, D. L. (2013). The genetic causes of convergent evolution. Nature Reviews Genetics, 14(11), 751–764. 10.1038/nrg3483 24105273

[ece35329-bib-0076] Strandh, M. , Jönsson, J. , Madjidian, J. A. , Hansson, B. , & Lankinen, Å. (2017). Natural selection acts on floral traits associated with selfing rate among populations of mixed‐mating *Collinsia heterophylla* (Plantaginaceae). International Journal of Plant Sciences, 178(8), 594–606.

[ece35329-bib-0077] Toräng, P. , Vikström, L. , Wunder, J. , Wötzel, S. , Coupland, G. , & Ågren, J. (2017). Evolution of the selfing syndrome: Anther orientation and herkogamy together determine reproductive assurance in a self‐compatible plant. Evolution, 71(9), 2206–2218. 10.1111/evo.13308 28722132

[ece35329-bib-0078] USDA and NRCS (2017). The PLANTS database. Greensboro, NC: National Plant Data Team.

[ece35329-bib-0079] Vallejo‐Marin, M. , Walker, C. , Friston‐Reilly, P. , Solis‐Montero, L. , & Igic, B. (2014). Recurrent modification of floral morphology in heterantherous *Solanum* reveals a parallel shift in reproductive strategy. Philosophical Transactions of the Royal Society B: Biological Sciences, 369(20130256). 10.1098/rstb.2013.0256 PMC4084541 25002701

[ece35329-bib-0080] Van der Auwera, G. A. , Carneiro, M. O. , Hartl, C. , Poplin, R. , delAngel, G. , Levy‐Moonshine, A. , … DePristo, M. A. (2013). From FastQ data to high‐confidence variant calls: The genome analysis toolkit best practices pipeline. Current Protocols in Bioinformatics, 43, 1–33. 25431634 10.1002/0471250953.bi1110s43 PMC4243306

[ece35329-bib-0081] Weir, B. S. , & Cockerham, C. C. (1984). Estimating F‐statistics for the analysis of population structure. Evolution, 38(6), 1358–1370. 28563791 10.1111/j.1558-5646.1984.tb05657.x

[ece35329-bib-0082] Whitlock, M. C. (2008). Evolutionary inference from QST. Molecular Ecology, 17(8), 1885–1896. 18363667 10.1111/j.1365-294X.2008.03712.x

[ece35329-bib-0083] Whitlock, M. C. , & Gilbert, K. J. (2012). QST in a hierarchically structured population. Molecular Ecology Resources, 12(3), 481–483. 10.1111/j.1755-0998.2012.03122.x 22336101

[ece35329-bib-0084] Whitlock, M. C. , & Guillaume, F. (2009). Testing for spatially divergent selection: Comparing QST to FST. Genetics, 183(3), 1055–1063. 19687138 10.1534/genetics.108.099812 PMC2778959

[ece35329-bib-0085] Willing, E. M. , Dreyer, C. , & van Oosterhout, C. (2012). Estimates of genetic differentiation measured by FST do not necessarily require large sample sizes when using many SNP markers. PLoS ONE, 7(8), 1–7. 10.1371/journal.pone.0042649 PMC3419229 22905157

[ece35329-bib-0086] Wilson, P. J. , Thompson, K. , & Hodgson, J. G. (1999). Specific leaf area and leaf dry matter content as alternative predictors of plant strategies. The New Phytologist, 143(1), 155–162. 10.1046/j.1469-8137.1999.00427.x

[ece35329-bib-0087] Worley, A. C. , & Barrett, S. C. (2000). Evolution of floral display in *Eichhornia paniculata* (Pontederiaceae): Direct and correlated responses to selection on flower size and number. Evolution, 54(5), 1533–1545. 10.1111/j.0014-3820.2000.tb00699.x 11108582

